# Dietary Components That May Influence the Disturbed Gut Microbiota in Chronic Kidney Disease

**DOI:** 10.3390/nu11030496

**Published:** 2019-02-27

**Authors:** Denise Mafra, Natália Borges, Livia Alvarenga, Marta Esgalhado, Ludmila Cardozo, Bengt Lindholm, Peter Stenvinkel

**Affiliations:** 1 Post Graduation Program in Medical Sciences, Federal Fluminense University (UFF), Niterói-Rio de Janeiro (RJ) 24220-900, Brazil; liviaalvarenga92@gmail.com; 2 Post Graduation Program in Cardiovascular Sciences, Federal Fluminense University (UFF), Niterói-Rio de Janeiro (RJ) 24220-900, Brazil; nat_borges_@hotmail.com (N.B.); martaesgalhado@hotmail.com (M.E.); ludmila.cardozo@gmail.com (L.C.); 3 Division of Renal Medicine and Baxter Novum, Department of Clinical Science, Technology and Intervention, Karolinska Institutet, SE-171 77 Stockholm, Sweden; Bengt.Lindholm@ki.se

**Keywords:** gut microbiota, diet, nutrients, chronic kidney disease

## Abstract

Gut microbiota imbalance is common in patients with chronic kidney disease (CKD) and associates with factors such as increased circulating levels of gut-derived uremic toxins, inflammation, and oxidative stress, which are linked to cardiovascular disease and increased morbimortality. Different nutritional strategies have been proposed to modulate gut microbiota, and could potentially be used to reduce dysbiosis in CKD. Nutrients like proteins, fibers, probiotics, and synbiotics are important determinants of the composition of gut microbiota and specific bioactive compounds such as polyphenols present in nuts, berries. and fruits, and curcumin, may also play a key role in this regard. However, so far, there are few studies on dietary components influencing the gut microbiota in CKD, and it is therefore not possible to conclude which nutrients should be prioritized in the diet of patients with CKD. In this review, we discuss some nutrients, diet patterns and bioactive compounds that may be involved in the modulation of gut microbiota in CKD and provide the background and rationale for studies exploring whether nutritional interventions with these dietary components could be used to alleviate the gut dysbiosis in patients with CKD.

## 1. Introduction

The human gut ecosystem is very complex, supporting a diverse and dynamic bacterial community, which is of paramount importance to the host´s health because gut microbiota has no simple commensal relationship with humans, but rather takes part in a mutualistic symbiotic cooperation offering benefits for all involved [1]. Under normal conditions, the composition and functions of gut microbiota differ according to the physiological state of the host with major changes taking place already before we are born; in the pre- and postnatal environment, determinants of gut microbiota like gestational age, type of delivery, and breast-feeding have long lasting effects on the gut microbiota profile for years to come [2].

Obesity, diabetes mellitus, cardiovascular disease (CVD), Alzheimer’s, and chronic kidney disease (CKD) are all associated with factors contributing to *dysbiosis* [3]. In advanced CKD, uremia alters the biochemical milieu, promoting disturbances in gut microbiota and the intestinal barrier [4,5]. In addition to uremia, accumulation of metabolites such as uric acid, inadequate intake of fiber (due to restrictions of fruits and vegetables required for preventing hyperkalemia), and multi-drug regimens alter the biochemical environment in the uremic intestines contributing to dysbiosis [5]. 

The presence of dysbiosis favors the growth of bacteria that possess enzymes capable of generating uremic toxins such as indoxyl sulfate (IS), p-cresyl sulfate (p-CS), indole-3-acetic acid (IAA), and trimethylamine-N-oxide (TMAO), which accumulate in CKD [6]. In addition, dysbiosis causes the breakdown of epithelial tight junctions, leading to translocation of lipopolysaccharides (LPS), immune dysregulation, and inflammation [7]. These alterations in the uremic milieu associate with severe consequences such as further progression of CKD towards end stage renal disease, complications such as protein energy wasting, and CVD, ultimately leading to increased mortality [8,9].

Several factors influence the diversity of gut microbiota, including genetics, comorbidities and environmental factors such as exercise, smoking, and intake of drugs; however, the diet, food patterns and specific dietary components, is no doubt a primary driver of the composition of gut microbiota; i.e., microbes that are not digested in the stomach or absorbed in the small intestines and therefore are able to colonize the colon [10]. Moreover, the composition of the diet, and lack or surplus of specific nutrients, are key determinants of the generation rate and effects of metabolites produced from these bacteria [11]. 

Several therapeutic strategies involving nutritional compounds have been proposed to improve uremic dysbiosis, such as *probiotics* [7,12,13], *prebiotics* [14,15,16], *synbiotics* [17], *bioactive compounds* [18,19], and *low protein diets* [20]. The purpose of this review is to discuss nutritional interventions that potentially could benefit CKD patients through modulation of gut microbiota.

## 2. Probiotics

Probiotics have emerged as a promising adjuvant therapy in CKD. Probiotics are defined by the FAO and WHO as “*live microorganisms that, when administered in adequate amounts, confer a health benefit on the host*” [21]. Mechanisms of the action of probiotics include production of bacteriocins, competition with pathogenic bacteria for nutrients, blocking of adhesion sites for pathogenic bacteria, maintaining the integrity of the intestinal barrier, and modulation of immune response [22]. Examples of clinical applications of probiotics in CKD are presented in Table 1.

Main outcome measures have included blood urea nitrogen (BUN), uremic toxins such as IS, p-CS, IAA and TMAO, and inflammatory markers. Although the effects of interventions with probiotics have been inconsistent, some have reported that probiotic supplementation decreased BUN [12,23]. Probiotics have been thought to reduce urea levels because certain microorganisms synthesize urease, which hydrolyzes urea in the gut lumen, thereby lowering plasma levels of urea. In contrast, we observed an increase in BUN after probiotic treatment in hemodialysis (HD) patients. This may be due to a vicious cycle where the high intraluminal urea concentration in which probiotic strains were introduced favors growth of bacteria with urease activity, which, via generation of ammonia, a substrate for increased production of urea in the liver, may lead to a further increase of plasma BUN levels [13]. Also, since hydrolysis of urea leads to subsequent production of ammonium that in part is absorbed, returned to the liver (and metabolized again into urea from urea cycle), and converted to ammonium hydroxide, which is harmful to epithelial barrier, this may contribute to increased intestinal barrier permeability [5,23,24].

Studies also show alterations in levels of gut-derived uremic toxins after probiotic intervention. Taki et al. [25] showed reduction in IS plasma levels consistent with Eidi et al. [26] who observed a decrease in the serum levels of the uremic toxins, phenol, and p-CS. In contrast, we showed no appreciable results after a probiotic intervention in HD patients; there was an increase in IS levels, but no changes in p-CS or IAA levels. In non-dialysis CKD patients, we observed that the same probiotic treatment was unable to change IS, p-CS, IAA, and TMAO [13,24]. The conflicting findings in the literature are reflected in a recent meta-analysis [27], which showed no effects of probiotics on uremic toxins.

Few studies have evaluated the impact of probiotics on gut microbiota composition. It is of interest that the effects of probiotics may be a result of the influence of probiotics on metabolic capacities of the resident bacteria, modulating the gut microbiota transcriptome [32]. Gut microbiota transcriptome refers to bacterial gene expression, offering a more informative perspective about gut microbiota functionality. Therefore, the effects of probiotics are not necessarily conditioned by changes in the gut microbiota profile [32]. In addition to modulation of the generation of metabolites from gut microbiota, probiotics also play an important role in the host immune response by regulating gene expression and signaling pathways in the host cells [33]. Although biomarkers of inflammation have been assessed in several studies on probiotics, the results have not been consistent [9,34]. Wang et al. [29] observed a decrease in serum levels of the pro-inflammatory cytokines tumor necrosis factor, interleukin (IL)-5, IL-6, endotoxins and an increase in IL-10 after probiotic treatment, while Barros et al. [24] showed an increase in IL-6 plasma levels after probiotic supplementation. On the other hand, others did not find any significant effects of probiotics on inflammatory markers [13,28,30,31]. As in vitro studies show that different probiotic strains may promote different results by changing the balance between production of pro-inflammatory and anti-inflammatory cytokines, this may explain the discrepant results [35,36,37]. It is important to keep in mind that probiotics may not always be beneficial, especially in sick patients in whom various pathological processes affect the gut environment. Whether to use a single strain or a combination of probiotic strains also remains a matter for discussion because bacteria may behave differently when administered in combinations rather than in isolated form [38]. 

Since no beneficial effects of probiotics have been demonstrated in CKD, prescription of probiotics as a sole intervention in these patients is not recommended. However, further studies should assess if the combination of probiotics with a healthy diet, containing components like prebiotics and bioactive compounds, together with a healthy life style, could benefit the gut ecosystem and consequently the host [4,13,32].

## 3. Paraprobiotics and Postbiotics

With the advancement of knowledge regarding mechanisms of modulation of gut microbiota, it has been recently suggested that bacterial viability is not necessary in the therapy that aims to promote benefits for individuals. New approaches have been suggested, such as *paraprobiotics*, that refers to inactivated microbial cells, and *postbiotics*, that refers to soluble factors secreted by live bacteria or released after bacterial lysis (SCFA, enzymes, peptides, peptidoglycan-derived muropeptides, cell surface proteins, vitamins, plasmalogens, and organic acids) [39].

One advantage of using paraprobiotics and postbiotics is that these interventions may be safer than probiotics because they reduce the risk of microbial translocation, infection, or enhanced inflammatory responses [39]. The mechanisms involved, and the possible benefits achieved, by these approaches need to be elucidated, but it is likely that they may contribute to improve the intestinal homeostasis and host metabolism, which would be interesting for CKD patients. The use of foods as a vehicle for paraprobiotics and postbiotics seems promising, but needs to be further explored and validated clinically [40,41].

## 4. Prebiotics

Prebiotics are non-digestible food ingredients, such as dietary fibers, different oligo- and polysaccharides, and resistant starches that selectively change the composition or activity of the gut microbiota in a way that benefits the host [42]. Prebiotics are found naturally in many fruits, cereals, vegetables (asparagus, sugar beet, garlic, chicory, onion, banana), honey and in human milk [1,43,44]. The bacterial fermentation of prebiotics stimulates the growth of certain colonic bacteria, particularly Bifidobacteria and Lactobacilli species, at the cost of other strains of bacteria, like Bacteroides, Clostridia and Enterobacteria [45]. As a result of the fermentation of prebiotics to short chain fatty acids (SCFA), particularly acetate, propionate and butyrate, prebiotics have been shown to (1) improve glycemia and insulin resistance; (2) improve dyslipidemia; (3) reduce hunger; (4) reduce colonic pH; (5) attenuate immunomodulatory effects; (6) reduce macronutrient digestion; (7) delay gastric emptying and/or intestinal transit time; (8) reestablish gut barrier integrity; (9) reduce the exposure to lipopolysaccharides (LPS) and uremic toxins; and (10) reduce oxidative stress and inflammation [16,43,46,47].

Despite abundant documentation in the literature about these positive effects, little is known about the clinical effects of prebiotics in CKD. In CKD rats fed a high amylose resistant starch diet, a decrease in microbial diversity and an increase in the amount of Bifidobacteria and Bacteroidetes-to-Firmicutes ratio were observed [48]. In another study in CKD mice, guar gum increased the Lactobacillus counts [49]. Moreover, xylooligosaccharide decreased six out of the nine CKD-associated bacterial genera in CKD mice [50]. In agreement, Tayebi-Khosroshahi et al. [51] observed an increase in Bifidobacteria and Lactobacillus counts in patients with CKD stage 3-4 after lactulose supplementation. Most animal and human studies on prebiotics in CKD investigated only the effects on uremic toxin levels [50,52,53]. In CKD animals fed with resistant starch [48], arabino-xylo-oligosaccharide [54], or gum acacia [55] or xylooligosaccharide [50], a reduction in IS and/or p-CS was observed.

In HD patients, an oligofructose-enriched inulin dietary supplementation reduced the generation rates and serum concentrations of p-CS, but had no effect on IS [56]. Partly in contrast, high amylose resistant starch supplementation reduced serum IS and showed a trend towards a reduction of p-CS in HD patients [52]. In accordance, we reported that resistant starch supplementation reduced the IS plasma levels in HD patients [16]. In CKD stage 3–5, supplementation of muffins containing pea hull fiber, together with inulin supplementation reduced plasma levels of p-CS [53]. In another study in CKD stage 3–5, supplementation with arabinoxylan oligosaccharides decreased the levels of TMAO, but had no effect on IS or p-CS levels [57]. 

The observed reduction in plasma levels of uremic toxins following these interventions possibly occurs by modulation of the gut microbiota, by decrease of the bacteria that produce its precursors and by restoring the integrity of the intestinal barrier, which reduces translocation of toxins into the bloodstream. Indeed, in several studies supplementation of prebiotics in CKD resulted in a reestablishment of the gut microbiota symbiosis and a restored integrity of the gut barrier [14,48,49,51]. These effects may be related to a reduction of markers of inflammation and oxidative stress in CKD [14,51,55]. Prebiotics may also exert other potentially beneficial effects in CKD, such as improved glucose and lipid profiles [54]. Others have observed a reduction of BUN [56], improved estimated glomerular filtration rate (eGFR) [53], increased fecal nitrogen excretion [58], and increased generation of SCFA [49,50]. Taken together, since the current literature suggests that prebiotics have favorable effects in CKD (Table 2), CKD patients should be encouraged to increase their intake of foods with a high content of prebiotics. Prebiotics, especially those found naturally in foods, can be of great advantage even in the early stages of CKD, in order to improve intestinal health, restore a more healthy metabolic profile, mitigate the comorbidities effects, thus slowing the progression of CKD.

## 5. Synbiotics

The combination of pro- and pre-biotics is called synbiotics. In 1995, Gibson and Roberfroid defined synbiotics as *“a mixture of probiotics and prebiotics that beneficially affects the host by improving the survival and implantation of live microbial dietary supplements in the gastrointestinal tract, by selectively stimulating the growth and/or activating the metabolism of one or a limited number of health-promoting bacteria, and thus improving host welfare”* [60]. The main purpose of this combination is to improve the survival of probiotic microorganisms while selectively stimulating the proliferation of health promoting bacteria in the gastrointestinal tract [61]. Synbiotics may also be used in specific products in which the prebiotic component selectively favors the growth of the probiotic component [62,63]. The use of synbiotics is thought to have better health promoting effects compared with isolated use of pre- or probiotics [60,62]. The health effects of synbiotics depend on a combination of characteristics of the selected probiotics and prebiotics [63]. One commonly used synbiotic combination of prebiotics and probiotics comprises bacteria of the genus Bifidobacterium or Lactobacillus with fructooligosaccharides (FOS), which have the potential to stimulate growth of these strains [1,61,62].

As mentioned above, the use of probiotics alone in CKD patients is not effective to modulate systemic inflammation, uremic toxins and biochemical parameters [64]. Whether the symbiotic combination of pro- and prebiotics, or prebiotics alone may be the best therapeutic strategy in CKD is not yet known, as RCTs comparing the effects of synbiotics vs prebiotics have not been conducted. In 2014, Cruz-Mora et al. [65] observed that two months of synbiotic supplementation (*Lactobacillus acidophilus* and *Bifidobacterium bifidum* in addition to inulin) altered the intestinal microbiota by increasing the count of Bifidobacteria in HD patients. Rossi et al. [17] confirmed this finding as they observed alterations of the stool microbiome, specifically with an increase of *Bifidobacterium* and depletion of *Ruminococcaceae*. As seen in Table 3, use of synbiotics seems to reduce BUN, improve gastrointestinal symptoms [65,66,67] and delay the decline of GFR in non-dialysis CKD patients [68]. With regard to uremic toxins, the use of synbiotics deserves attention since this intervention may reduce circulating levels of p-cresol and p-CS [17,69,70].

## 6. Bioactive Compounds

Much recent attention has been paid to the bioactive compounds of foods, such as polyphenols, and their capacity to modulate intestinal microbiota. These compounds are potent antioxidants and natural anti-inflammatories and are extensively used for the prevention of chronic “burden of lifestyle diseases” related to inflammation and oxidative stress [18,19,71]. Studies in humans show that the bioactive compounds present in some foods, like grapes, red wine, pomegranate, garlic, green tea, chocolate, turmeric, and cranberry, modify the composition of human intestinal microbiota (Table 4). A breakthrough was recently made when Singh et al. [72] showed that Urolithin A, a major microbial metabolite derived from polyphenols of berries and pomegranate fruits, upregulated gut barrier epithelial tight junction proteins via the NRF2 pathway. Since Urolithin A activates the aryl hydrocarbon receptor (AhR), and IS antagonizes AhR, it would be of outmost interest to conduct a long-term RCT with berries in CKD 4–5 patients and to study the effects on levels of uremic toxins and inflammation markers. However, to the best of our knowledge, no study has evaluated the effects of any of these bioactive compounds on the gut microbiota profile or uremic toxin levels in CKD. Indirect support that a beneficial effect could be expected in such trials comes from a study showing that intake of pomegranate juice attenuated the increase in oxidative stress induced by intravenous iron in HD patients [72]. 

Anhê et al. [73] showed that cranberry extract modulates the gut microbiota of rats with metabolic syndrome by increasing the proportion of the mucin-degrading bacterium *Akkermansia* species. In 2016, they proposed a hypothesis about the possible mechanisms involved in modulation in the Akkermansia bacteria by cranberry [74]. They showed that cranberry extract with high content of protocyanidins, a class of polyphenols with a high molecular weight, favors increased secretion of mucus, creating a favorable environment for Akkermansia [74]. These same mechanism(s) may be valid for other compounds, such as anthocyanins, catechins, resveratrol, quercetin and tannins [75,76,77,78]. 

Other studies propose that anthocyanins present in fruits, such as açaí, blackberry, strawberries, red grapes and, cherries also modulate gut microbiota. Anthocyanins are absorbed through the gastrointestinal tract, especially in the stomach, and at least 75% of the anthocyanins reach the colon where this compound is biotransformed by the action of enteric bacteria on phenolic degradation products [79], and modulate the gut microbiota [78,80]. 

Curcumin, a bioactive polyphenol of turmeric, may also modulate gut microbiota in CKD. Curcumin blocks the excessive production of LPS, avoiding breakdown of the intestinal barrier, the circulation of LPS, and the production of proinflammatory cytokines, such as TNF and IL-1. Finally, curcumin may modulate the production of intestinal alkaline phosphatase (iALP), an enzyme participating in the first line of defense in the intestinal lumen [81]. Shen et al. [82] observed that curcumin modulates the gut microbiota by modifying the bacterial communities present in the intestines, including *Prevotellaceae, Bacteroidaceae* and *Rikenellaceae*. In addition, curcumin modulated the gut microbiota by increasing bacterial-producing butyrate species *Clostridium cluster IV* and *Clostridium* subclusters [83].

To date, there are no in vitro or in vivo studies showing the effects of bioactive compounds on gut microbiota modulation in CKD. However, studies in other populations suggest that bioactive compounds can modulate the uremic gut microbiota. In this context, our hypothesis would be that the bioactive compounds of foods could be beneficial for the modulation of microbiota both by promoting colonization by beneficial bacteria and by reduction of oxidative stress and inflammation.

## 7. Low Protein Diet 

Some studies have evaluated the effects of a low protein diet (LPD) on gut microbiota in CKD. According to guidelines, a restricted protein intake, 0.6–0.75 g/kg/day of protein, is recommended for non-dialysis CKD patients [93,94]. According to one hypothesis [93], the LPD prescribed to non-dialysis CKD patients could–in addition to preserving renal function–be an important strategy to decrease levels of uremic toxins. In fact, Marzocco et al. [95] showed that a very low protein diet (0.3 g/kg/day) supplemented with ketoanalogues, prescribed in predialysis CKD patients, reduced IS serum levels by 37% when compared with a LPD after only one week of treatment, thus corroborating the above mentioned hypothesis about LPD. Black et al. (20) observed that non-dialysis CKD patients who received LPD for 6 months presented significant reduction in p-CS serum levels, and also induced changes in gut microbiota.

## 8. Dietary Patterns and Microbiota

Due to the synergistic effects of different components in food, recent studies increasingly focus on the assessment of whole eating patterns instead of individual nutrients. Studies show that healthy dietary patterns, (rich in fruits and vegetables, fish, whole grain, and fiber, and reduced in red meat, sodium, and refined sugar) associate with a reduced risk of all-cause mortality in CKD [96].

There are many studies demonstrating that the type and amount of ingested food may impact on the profile of gut microbiota and metabolites produced in the gut (Table 5). Some studies showed that vegetable-based proteins (such as soybeans, beans, chickpeas, peas, and lentils, among other sources) have different renal effects compared to animal-derived proteins [96,97]. In addition, animal-proteins seem to have a greater impact on altering the gut microbiota than vegetable-proteins [98].

Several studies confirm that protein intake increases the production of uremic toxins by gut microbiota [93,99,100]. In fact, red meat consumption contributes to a high production of uremic toxins by the gut microbiota, such as TMAO, IS, and p-CS [15,101], which are uremic toxins associated with an increased risk of CVD [102]. Reducing red meat intake in CKD patients may therefore be a good strategy to reduce cardiovascular risk, as well as decrease the rate of progression of CKD [101]. Therefore, according to available evidence [101], the consumption of red meat should be controlled in CKD patients and the largest amounts of proteins should instead come from non-animal proteins. Indeed, Patel et al. [100] reported that in subjects with normal renal function, p-CS and IS production was on average 60% lower in vegetarians than in participants who ate an unrestricted diet. Indeed, a cross-sectional study showed that vegetarian HD patients had lower IS and p-CS levels when compared with HD patients who were omnivores [99]. The benefits of a vegetarian diet in reducing uremic toxins may be due to increased fiber intake and a lower protein intake provided by such a diet. It is important to consider that the benefits of the vegetable-based protein diet are probably related not only to protein but could be attributed to all other components of this dietary pattern. Despite the benefits associated with a vegetable-based protein diet, it is not yet known whether this diet pattern could promote more benefits to the gut microbiota than other patterns, and thereby perhaps render long-term health advantages to CKD patients [102,103]. The generation of uremic toxins is directly associated with the ratio of protein-to-carbohydrate intake because a high ratio favors the prevalence of proteolytic bacteria over saccharolytic species. In this context, the Mediterranean diet may be a strategy for CKD patients to modulate the gut microbiota, because it is based on a considerable consumption of carbohydrates, whole grain foods rich in fiber and nuts, high quantities of fruits and vegetables, moderate consumption of fish, poultry and dairy, low consumption of red meat products, use of olive oil as the main source of fat, and regular but moderate wine consumption. This healthy dietary pattern favors the prevalence of saccharolytic species, the production of SCFAs, and increases the “good” microbiota with high proportions of *Bifidobacteria, Lactobacilli, Eubacteria, Bacteroides* and *Prevotella* [104,105]. On the other hand, the typical Western diet, characterized by an over-consumption of refined sugars, animal fat and protein, salt, saturated fat, and a low intake of fiber, may be detrimental for the gut microbiota profile, favoring proteolytic bacteria, and generate more dysbiosis [95].

Nuts are important components of the Mediterranean diet, and their health benefits are associated with their fatty acid profile and high content of protein, fiber, vitamin, phytosterols, and phenolics. Besides, nuts have prebiotic properties due to their content of fibers and polymerized polyphenols components, which are metabolized by gut microbiota forming bioactive metabolites and modulating the microbiota profile [106].

In addition to anti-oxidant, anti-inflammatory and antimicrobial effects attributed to **olive oil**, their phenolic compounds appear to influence gut microbiota, and it has been suggested that cardiovascular benefits attributed to olive oil may partly be due to a positive effect on gut bacteria. Phenolic compounds can selectively stimulate the growth of *Lactobacillus* that are involved in lipid metabolism. A proposed mechanism is that these bacteria possess bile-salt hydrolase that deconjugates bile acids, preventing them from being reabsorbed, resulting in the excretion of free bile acids and cholesterol in feces. As a compensation, the hepatic uptake of LDL is upregulated and cholesterol levels decrease [107].

Some studies have evaluated the effects of a Mediterranean diet as compared to a less healthy, usual diet on gut microbiota [96,108,109] (Table 5). De Filippis et al. [108] evaluated 153 healthy individuals in a cross-sectional study and observed that most vegans and vegetarians, but only 30% of omnivore individuals, adhered to a Mediterranean diet. Besides, vegetarians and vegans had lower urinary TMAO levels. High-level adherence to a Mediterranean diet showed associations between consumption of vegetable-based diets and higher fecal levels of SCFA [96,108], higher *Prevotella* and fibre-degrading *Firmicutes* bacteria [95]. Also, a high adherence to the Mediterranean diet reduced *E. coli* counts and increased the *Bifidobacteria/E. coli* ratio [109]. According to Montemurno et al. [105], a Mediterranean diet represents an ideal dietary pattern for CKD patients. However, its effects on the gut microbiota of patients with peculiarities typical of CKD need to be investigated.

## 9. Other Diet Components

Although there are essentially no studies in CKD on the components of the diets described below, it is reasonable to assume that these foods, commonly used every day by people, irrespective of being a CKD patient or a healthy individual, may be health promoting. The fermentation of food with bacteria or yeast provides an anaerobic complex pathway, during which sugars are transformed by fermentation into ethanol and CO_2_ (alcoholic fermentation) or lactic acid. The lactic acid bacteria produce foods, such as cheese, soymilk, yoghurt, pickles, bread, etc, and the alcoholic process produce wine and beers [110,111]. 

Fermented food has prebiotic, probiotic, and biogenic (vitamins, lactotripeptides, polyamines, bacteriocins, polyphenols and gamma-aminobutyric acid) effects and increasing the intake of fermented food can therefore be a strategy to modulate gut microbiota imbalance. Although research on health promoting effects of fermented foods in CKD is limited, there is increased awareness of the beneficial impact of these foods on general health. Indeed, radical changes in food preservation with an absence of alkyl catechols (such as 4-vinylcatechol and 4-ethylcatechol) found in traditionally fermented food in the modern Western diet has negative consequences for NRF2-mediated cell defences [112]. As an example, pyranoanthocyanidins and 3-p present in the Western diet as an ingredient in ultra-processed foods, can affect the composition and functionality of intestinal microbiota, promoting negative effects on glucose metabolism and contributing to obesity [113]. In mice, it was observed that high sugar intake caused loss of microbial diversity, favoring bacteria capable of metabolizing such sugars, which are not common in the distal colon. As a result, intestinal microbiota may become capable of increasing the energy harvest from the diet [114].

In a systematic review, researchers observed that artificial sweeteners, such as saccharin, sucralose, aspartame, and acesulfame K, may modify the gut microbiota profile, leading to glucose intolerance and decreased visceral motility [115]. Artificial sweeteners inhibit the anaerobic fermentation of glucose and increase *Firmicutes*, altering the gut microflora. However, the pathways by which these artificial sweeteners alter the microbiota warrant further investigation [116,117]. Although intolerances are not always clinically verified, the gluten-free diet have become popular and is a new dietary trend. Supporters of this diet argue that gluten leads to inflammation and obesity [118]. However, gluten can be beneficial to the gut microbiota and a gluten-free diet may increase the population of unhealthy gut bacteria [119,120]. 

Since most bacteria from the gut need iron for their replication and survival, the availability of iron in the gut can alter the microbiota profile. Oral iron supplementation may cause growth of opportunistic pathogens such as *Salmonella, Shigella,* and *Campylobacter* that disturb gut microbiota composition, while other bacteria like *Lactobacillus* and *Bifidobacterium* require little (or no) iron to grow [121,122]. As anemia is highly prevalent and CKD patients are often treated with iron supplementation, Kortman et al. [123] have discussed the adverse effects of oral iron supplementation on the uremic gut microbiota. In fact, comparing pediatric CKD patients on HD and peritoneal dialysis (PD) treatment, PD patients exhibited an increased relative abundance of *Proteobacteria*; the authors proposed that this may be due to oral iron supplementation [124]. However, the potential effects of iron supplementation on the gut microbiota in CKD are still not known.

Calcium and magnesium may also have effects on the gut microbiota. Crowley et al. [125] observed that dietary supplementation with a marine multi-mineral blend (rich in calcium and magnesium) resulted in a significant increase in gut microbial diversity in adult male rats. Calcium may have beneficial effects on bacteria in the gut of obese animals [126]. However, to date, there is little evidence on the effects of magnesium and calcium supplementation on gut microbial diversity.

Phosphorus is important for gut microbiota metabolism, and as most patients with advanced CKD have high circulating levels of phosphorus that associate with various complications, phosphate-binding agents are used in most patients. These agents form phosphate complexes in the colon that change the intestinal microenvironment. Miao et al. [127] observed a reduction on microbial genera and decreased microbial diversity after the use of phosphate binders in HD patients while Lau et al. [128] showed that ferric citrate treatment increased bacterial diversity in CKD rats. Taken together, these studies point to a need to assess more comprehensively the effects of different long-term phosphate binders on gut microbiota in CKD patients. 

Gut microbiota have an essential role in vitamin production. However, the effects of vitamin supplements on metabolism in the gut are largely unknown. Nowadays, people are advised to supplement their diets with high doses of vitamins and studies have shown that this may change gut microbiota composition. Xu et al. [129] observed that after seven days with methylcobalamin and cyanocobalamin supplementation, total bacteria counts and the diversity of colon flora were reduced in cobalamin deficient patients. Vitamin A may also play a role in gut microbiota modulation [130]. However, the effects of vitamin supplements on the metabolism in the gut are largely unknown.

Vitamin D is one actor in the complex relationship between gut microbiota and the immune system. Vitamin D participates in the formation and function of the intestinal epithelium barrier and participates in the modulation of the intestinal immune system; thus, low levels of vitamin D can lead to increased intestinal permeability and, consequently, to a state of chronic low-grade inflammation. In addition, vitamin D supplementation may influence the composition of gut microbiota, since vitamin D may enhance the ability of macrophages to kill microbes such as *Escherichia coli* [131].

There are many non-nutritional bioactive compounds that provide protective and regenerative effects for the kidneys, and which may be used for the treatment of renal disease [132]. However, we do not include possible interactions and synergies of nutritional interventions with non-nutritional bioactive compounds on microbiota in the present review, which focuses on the role of dietary components.

## 10. Conclusions

**Figure 1:**
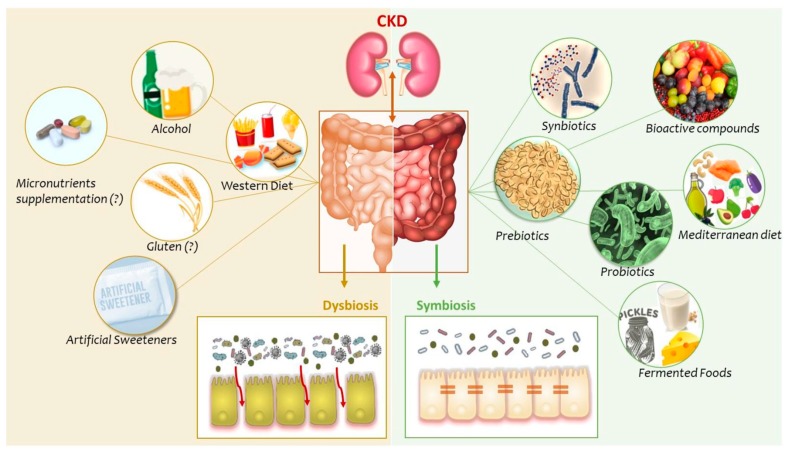
Dietary components and gut microbiota in chronic kidney disease (CKD). In CKD, several factors lead to alterations in composition and function of the gut microbiota. These disorders are associated with severe consequences for CKD patients, as part of a bidirectional gut-kidney axis. Here we present some dietary components that may modulate gut microbiota composition and their metabolites, contributing to dysbiosis or symbiosis.

Our diet is the most important, easily controlled factor that can modulate gut microbiota. Here, we reviewed studies that investigated how different dietary components may impact gut microbiota, its metabolites, and how this could influence the uremic phenotype in a positive way (Figure 1). To date, few studies have been conducted on the effects of dietary components on the gut microbiota profile in CKD, and most questions are still unanswered; for example, if the associations between gut dysbiosis and the uremic milieu are uni- or bidirectional. Despite emerging knowledge that gut microbiota is severely altered in the uremic milieu and that this may have profound consequences in terms of increased morbimortality and impaired quality of life, there is a conspicuous lack of studies that could tell us if gut dysbiosis can be treated or prevented, and whether this would improve the survival and well-being of patients. When planning for interventional studies, it is important to consider that the mechanisms involved in gut dysbiosis are complex, multifactorial, and interdependent. Therefore, an isolated intervention of one food item may not be effective; there is no *silver bullet* solution. According to the view of thinking that is now emerging, a whole set of measures acting synergistically are necessary to restore the composition and functionality of gut microbiota in CKD. Furthermore, since gut microbiota is specific to each person, nutritional interventions should be individualized and based on each person’s microbiota characteristics. While this kind of knowledge is not yet available, the rapid progress in diagnostic abilities based on genetic analyses of circulating microbial DNA may soon open new routes for interventional preventive and therapeutic strategies. Meanwhile, given the enormous potential of “food as medicine” in CKD, the impact of food and nutraceuticals on the uremic phenotype and its relation to gut dysbiosis deserves much more attention. The long-term effects of polypharmacy and specific drugs affecting the microbiome, such as phosphate binders, omeprazole and iron, as well as life style patterns including physical activity, are but a few of many important areas that also deserve further studies. 

## Figures and Tables

**Table 1:** Summary of studies involving probiotics interventions in CKD patients.

References	Study Design, Sample, Follow-up	Intervention	Results
Taki et al., 2005 [25]	Non-controlled trial; 27 HD patients; 3 months	3 × 10^9^ CFU/day of *B. longum* for 4 weeks, 6.0 × 10^9^ CFU/day from 4th to 8th week, 12.0 × 10^9^ CFU/day from 8th to 12th week.	↓ IS, homocystein and triglyceride serum levels ↑ folate serum levels
Ranganathan et al., 2010 [12]	RCT with crossover, multicenter; 46 non-dialysis CKD patients; 6 months	9 × 10^10^ CFU/day of a probiotic mix: *S. thermophilus, L. acidophilus, and B. longum*	↓ urea serum levels Overall improvement in quality of life ↔ Cr and or uric acid
Alatriste et al., 2014 [23]	RCT; 30 non-dialysis CKD patients; 2 months	8 × 10^9^ CFU/day vs. 16 × 10^9^ CFU/day of *L. casei* Shirota	↓ serum urea – in dose of 16 × 10^9^ CFU
Natarajan et al., 2014 [28]	RCT with crossover; 22 HD patients; 6 months	1.8 × 10^11^ CFU/day of a probiotic mix: *S. thermophilus, L. acidophilus, and B. longum*	↔ Uremic toxins or inflammatory markers.
Wang et al., 2015 [29]	Randomized, double-blind, placebo-controlled clinical trial; 39 peritoneal dialysis patients;6 months	10^9^ CFU/day of *B. bifidum*, 10^9^ CFU/day of *B. catenulatum*, 10^9^ CFU/day of *B. longum*, and 10^9^ CFU/day of *L. plantarum*	↓ serum TNF-α, IL-5, IL-6, and endotoxin ↑ serum IL-10
Soleimani et al., 2016 [30]	RCT; 60 diabetic patients on HD; 3 months	2 × 10^9^ CFU. *L. acidophilus, L. casei and B. bifidum*	↓ plasma glucose, serum insulin, HOMA-IR, HOMA-B, HbA1c, hs-CRP, MDA, SGA scores, TIBC ↑ plasma total antioxidant capacity ↔ lipid profiles, biomarkers of inflammation and oxidative stress
Shariaty et al., 2017 [31]	RCT; 34 HD patients; 3 months	3 × 10^10^ CFU of *L. acidophilus*, 3 × 10^9^ CFU of *L. casei*, 7 × 10^9^ CFU of *L. rhamnosus*, 5 × 10^8^ CFU of *L. bulgaricus*, 2 × 10^10^ CFU of *B. breve*, 1 × 10^9^ CFU of *B. longum,* 3 × 10^8^ CFU of *S. thermophilus*	↔ Hb or CRP levels
Borges et al., 2017 [13]	RCT; 33 HD patients; 3 months	9 × 10^10^ CFU/day of a probiotic mix: *S. thermophilus, L. acidophilus, and B. longum*	↑ plasma IS, K, urea; ↓ fecal pH ↔ p-CS, IAA, inflammatory markers (CRP and IL-6) or gut microbiota profile
Barros et al., 2018 [24]	RCT; 22 non-dialysis CKD patients; 3 months	9 × 10^10^ CFU/day of a probiotic mix: *S. thermophilus, L. acidophilus, and B. longum*	↑ IL-6 plasma levels ↔ Uremic toxins (TMAO, IS, p-CS and IAA), CRP, LPS or calprotectin.
Eidi et al., 2018 [26]	RCT; 42 HD patients; 1 month	1.6 × 10^7^ CFU/day of *L. Rhamnosus*	↓ phenol and p-cresol serum levels

**Abbreviations:** HD: hemodialysis; CFU: colony-forming units; CKD: chronic kidney disease; RCT: Randomized clinical trial; Cr: creatinine; CRP: C-reactive protein; K: potassium; LPS: lipopolysaccharides; MDA: malondialdehyde; IS: indoxyl sulfate; p-CS: p-cresyl sulfate; IAA: indole-3-acetic acid; TMAO: trimethylamine-N-oxide; IL: Interleukin; HOMA-IR: homeostatic model assessment- insulin resistance; HOMA-B: homeostatic model assessment- beta; HbA1c: glycated hemoglobin; hs-CRP: high sensitivity C-reactive protein; SGA: saturated fatty acids; TIBC: total iron binding capacity; Hb: hemoglobin; ↔: no change; ↑: increase; ↓: decrease.

**Table 2:** Summary of human studies involving prebiotics interventions in CKD patients.

References	Study Design, Sample, Follow-up	Prebiotics	Results
Younes et al, 2006 [58]	RCT with crossover; 9 non-dialysis CKD patients; 5 weeks	40 g/day fermentable carbohydrate (25 g whole-meal bread + 4.5 g inulin + 10.5 g crude potato starch)	↑ stool weight, fecal and urinary urea, fiber intake No change in eGFR
Meijers et al, 2010 [56]	Non-randomized, single-center, open-label phase; 22 HD patients; 4 weeks	10–20 g/day of oligofructose-enriched inulin	↓ 20% serum p-CS, generation rate and BUN ↔ IS serum or generation rates
Sirich et al, 2014 [52]	RCR; 56 HD patients; 6 weeks	15 g/day of high-amylose corn starch	↓ IS and a trend to p-CS free plasma ↔ body weight, CRP
Salmean et al, 2015 [53]	Single-blind, placebo controlled; 13 non-dialysis CKD patients; 6 weeks	10 g/day of pea hull fiber (Best Pea Fiber; Best Cooking Pulses, Portage la Prairie, Manitoba, Canada) + 15 g/day inulin	↓ 20% total plasma p-CS, ↑ stool frequency, fiber intake ↔ CRP, cystatin C, BUN, ammonia, eGFR
Poesen et al, 2016 [57]	RCT with crossover; 40 non-dialysis CKD patients; 4 weeks	10 g twice/day of arabinoxylan oligosaccharide	↓serum TMAO ↔ IS, p-Cs, p-Cglucuronide and phenylacetylglutamine, HOMA-IR
Tayebi-Khosroshahi et al, 2016 [51]	RCT; 32 non-dialysis CKD patients; 8 weeks	30 mm thrice/day of lactulose syrup	↑ fecal bifidobacteria and lactobacillus counts ↓ Cr
Tayebi Khosroshahi et al, 2018 [59]	RCT; 46 HD patients; 4 weeks	20–25 g/day of high amylose maize resistant starch	↓ TNF-α, IL-6, MDA, severity constipation, serum urea and creatinine ↔ IL-1β, hs-CRP, total antioxidant activity
Esgalhado et al, 2018 [16]	RCT; 31 HD patients; 4 weeks	16 g/day resistant starch (Hi-Maize^®^ 260)	↓ IL-6, TBARS, IS and a trend to protein carbonylation; ↑ fiber intake ↔ CRP, p-CS

**Abbreviations:** HD: hemodialysis; CKD: chronic kidney disease; RCT: randomized clinical trial; Cr: creatinine; CRP: C-reactive protein; FOS: fructooligosaccharide; p-CS: p-cresyl sulfate; IAA: indole-3-acetic acid; IS: indoxyl sulfate; TMAO: trimethylamine-N-oxide; IL: Interleukin; HOMA-IR: homeostatic model assessment- insulin resistance; hs-CRP: high sensitivity C-reactive protein; ↔: no change; ↑: increase; ↓: decrease; eGFR: estimated glomerular filtration rate; BUN: Blood urea nitrogen; TNF: Factor de necrose tumoral; MDA: malondialdehyde.

**Table 3:** Summary of studies involving symbiotics interventions in CKD patients.

References	Study Design, Sample, Follow-up	Intervention	Results
Nakabayashi et al., 2011 [70]	Clinical trial; 9 HD patients; 2 weeks	1 × 10^8^ Lactobacillus casei strain Shirota and Bifidobacterium breve strain Yakult + 4 g of prebiotic: 1.67 g or more galacto-oligosaccharides and <1.36 g of lactose and monosaccharide	↓ serum p-CS levels ↔ phenol or IS
Cruz-Mora et al., 2014 [65]	RCT; 18 HD patients; 2 months	(Lactobacillus acidophilus and Bifidobacterium bifidum), for total as probiotic of 2.0 × 10^12^ CFU 2.31 g of a prebiotic fiber (inulin); 1.5 g of omega-3 fatty acids (eicosapentaenoic and docosahexaenoic acid) and vitamins (complex B, folic acid, ascorbic acid, and vitamin E)	↑ *Bifidobacterium* counts ↓Gastrointestinal symptoms scores
Guida et al, 2014 [69]	RCT; 30 non-dialyzed CKD patients; 1 month	5 × 10^9^ Lactobacillus plantarum, 2 × 10^9^ Lactobacillus casei subsp. rhamnosus and 2 × 10^9^ Lactobacillus gasseri, 1 × 10^9^ Bifidobacterium infantis and 1 × 10^9^ Bifidobacterium longum, 1 × 10^9^ Lactobacillus acidophilus, 1 × 10^9^ Lactobacillus salivarius and 1 × 10^9^ Lactobacillus sporogenes and 5 × 10^9^ Streptococcus thermophilus) + prebiotic: 2.2 g inulin and 1.3 g of tapioca-resistant starch	↓ plasma p-CS
Viramontes-Horner et al, 2015 [66]	RCT; 42 HD patients; 2 months	Symbiotic gel (containing Lactobacillus acidophilus NCFM and Bifidobacterium lactis Bi-07 for a total of 11 × 10^6^ CFU + 2.31 g of a prebiotic fiber inulin + 1.5 g of omega-3 fatty acids and vitamins of complex B, folic acid, ascorbic acid, and vitamin E)	↓ Episodes of vomit, heart- burn, and stomachache, gastrointestinal symptons. No change in the prevalence of malnutrition, CRP and TNFα levels
Dehghani et al., 2016 [67]	RCT; 66 non-dialysis CKD patients; 6 weeks	2 × 500 mg containing 7 strains of probiotics: *Lactobacilus casei*, *Lactobacilus acidophilus*, *Lactobacilus bulgarigus*, *Lactobacilus rhamnosus*, *Bifidobacterium breve*, *Bifidobacterium longum*, *Sterptococus thermophilus* + FOS	↓ blood urea nitrogen ↔ Cr, uric acid
Pavan, 2016 [68]	Prospective observational study with randomized control, open-label design; 24 non-dialysis CKD patients; 6 months	15 billion cells/cfU of each one: Streptococcus thermophiles, Lactobacilllus acidophilus, Bifidobacterium longum + 100 mg Fructooligosaccharides	↓ GFR
Rossi et al, 2016 [17]	RCT with crossover; 31 non-dialyzed CKD patients; 6 weeks	High–molecular weight inulin, FOS and GOS and the probiotic component including 9 different strains across the Lactobacillus, Bifidobacteria, and Streptococcus genera	↑Bifidobacterium and ↓ Ruminococcaceae ↓ p-CS ↑albuminuria by 38 mg/24 h ↔ IS, eGFR, IL-1b, IL-6, IL-10, TNF-a, serum oxidative stress biomarkers (F2- isoprostanes and glutathione peroxidase), and LPS

**Abbreviations:** HD: hemodialysis; CKD: chronic kidney disease; RCT: randomized clinical trial; Cr: creatinine; CRP: C-reactive protein; FOS: fructooligosaccharide; GOS: galacto-oligosaccharides; p-CS: p-cresyl sulfate; IS: indoxyl sulfate; IAA: indole-3-acetic acid; TMAO: trimethylamine-N-oxide; IL: Interleukin; CFU: colony-forming unit; TNF- a: TNF: Factor de necrose tumoral- alpha; GFR: glomerular filtration rate; eGFR: estimated glomerular filtration rate; LPS: lipopolysaccharide; ↔: no change; ↑: increase; ↓: decrease.

**Table 4:** Summary of human studies involving bioactive compounds and gut microbiota.

References	Study Design, Sample, Follow-up	Intervention	Results
Clavel et al. (2005) [84]	RCT; 39 postmenopausal women; 1 month	100 mg/day of isoflavones supplemented in cereal bars and gelified milk	↑ Lactobacillus-Enterococcus ↑ Faecalibacterium prausnitzii ↑ Bifidobacterium ↔ Atopobium ↔ Bacteroides
Queipo-Ortuno et al. (2012) [85]	RCT;10 healthy male volunteers; 20 days	Group 1: de-alcoholized red wine (272 mL/day) Group 2: red wine (272 mL/day) Group 3: gin (100 mL/day)	Group 1: ↑ Fusobacteria ↓ Bacteroidetes and Firmicutes Group 2: ↑ *Proteobacteria, Fusobacteria, Firmicutes, Bacteroidetes, Bacteroides, Prevotella* and the B. uniformis. Group 3: ↑ Clostridium and the Clostridium histolyticum; ↓ Prevotella; ↓ CRP; total cholesterol
Song et al. (2015) [86]	RCT; 28 obese women; 3 months	2 pouches in a day, equivalent of 6.7 g dried Schisandra chinensis fruit	↑ *Akkermansia, Roseburia, Bacteroides, Prevotella, and Bifidobacterium* ↓ *Ruminococcus* ↓ blood glucose, triglycerides
Eid et al. (2015) [87]	RCT; 21 healthy volunteers; 21 days	50 g of palm date	↔ growth of the faecal microbiota
Moreno-Indias et al. (2015) [88]	RCT; 10 metabolic syndrome in obese patients; 1 month	red wine (272 mL per day) or de-alcoholized red wine (272 mL per day)	Red wine and de-alcoholized red wine: ↓ Clostridium and the Clostridium histolyticum ↑ Blautia coccoides–Eubacterium rectale, Faecalibacterium prausnitzii, Roseburia and Lactobacillus ↓ LPS
Janssens et al. (2016) [89]	RCT; 58 Caucasian men and women; 3 months	green tea (>0.56 g/d epigallocatechin-gallate + 0.28*0.45 g/d caffeine) capsules	↔ growth of the faecal microbiota
Li et al. (2015) [75]	RCT; 20 healthy participants; 1 month	1g of pomegranate extract daily	↑ Actinobacteria, Butyrivibrio, Enterobacter, Escherichia, Lactobacillus, Prevotella, Serratia and Veillonella. ↓ Firmicutes and Collinsell
Barroso et al. (2017) [90]	RCT; 15 healthy volunteers; 28 days	250 mL of red wine per day	↑ Slackia, Gordonibacter, Oscillatoria, Veillonella and Oenococcus
Most et al. (2017) [91]	RCT; 37 overweight and obese men and women; 3 months	epigallocatechin-3-gallate (282 mg/day) and resveratrol (80 mg/day)	↓ Bacteroidetes and Faecalibacterium prausnitzii in men ↔ Bacteroidetes and Faecalibacterium prausnitzii in women
Peterson et al. (2018) [92]	RCT; 14 healthy volunteers; 2 months	Group 1: turmeric tablets contained 1000 mg turmeric root (Curcuma longa) plus 1.25 mg black pepper–derived extract of piperine alkaloid. Group 2: curcumin tablets contained 1000 mg of curcumin (Curcumin C3 Complex) plus 1.25 mg black pepper.	Group 1: ↑ in observed species 7% (156 vs. 167) ↓ Eisenbergiella tayi; ↑ Alistipes putredinis Group 2: ↑ 69% (127 vs. 215) in detected species ↓ Coprococcus catus; ↑ Raoultella electrica; (Clostridium) xylanolyticum; Collinsella aerofaciens; Kluyvera intermedia

**Abbreviations:** RCT: randomized, double-blind, placebo-controlled; CRP: C-reactive protein; LPS: lipopolysaccharides; ↔: no change; ↑: increase; ↓: decrease.

**Table 5:** Summary of human studies involving different diets and their effects on the gut microbiota.

References	Study Design, Sample, Follow-up	Intervention	Results
		**CKD patients**	
Marzocco et al., 2013 [95]	RCT with crossover; 32 non-dialysis CKD patients; 1 week	VLPD (0.3 g/kg bw/day) + ketoanalogues LPD (0.6 g/kg bw/day)	VLPD changed the IS level with a reduction of 37% when compared to LPD
Kandouz et al., 2016 [99]	Cross-sectional; 138 HD patients from a cohort were analyzed and 16 patients were strict vegetarians	Vegetarian diet	↓ IS and p-CS levels; ↓ serum urea, and phosphate and estimated urea nitrogen intake before HD
Black et al., 2018 [20]	Longitudinal; 30 non-dialysis CKD patients; 6 months	LPD (0.6 g/kg/day)	↓ p-CS plasma levels Change in the intestinal microbiota profile ↔ IS, IAA
Mafra et al, 2018 [101]	Prospective pilot study; 9 non-dialysis CKD patients; 6 months	LPD (0.6 g protein/kg day)	↓ TMAO plasma levels
Patel et al., 2012 [100]	15 healthy vegetarian individuals 11 health individuals – normal diet	Vegetarian diet	↓ p-CS and IS production rates
		**Non CKD patients**	
De Filippis et al, 2016 [108]	Cross-sectional survey; 51 vegetarians, 51 vegans, 51 omnivores	Adherence to the Mediterranean diet	Associations between consumption of vegetable-based diets and higher levels of short-chain fecal fatty acids, Prevotella and fiber-degrading Firmicutes; ↓ urinary TMAO levels in vegetarian and vegan diet
Mitsou et al, 2017 [109]	Cross-sectional study, 120 healthy participants	Adherence to the Mediterranean diet	↓ *Escherichia Coli* counts, ↑ Bifidobacteria
Garcia-Mantrana et al, 2018 [96]	Cross-sectional study; 27 healthy volunteers	Adherence to the Mediterranean diet	↑ Bifidobacterial counts, ↑ concentration of acetate, propionate, and butyrate in fecal samples, ↑ Bacteroidetes and a lower Firmicutes–Bacteroidetes ratio

**Abbreviations:** VLPD: very low-protein diet; LPD: Low-protein diet; p-CS: p-cresyl sulfate; IS: indoxyl sulfate; IAA: indole-3-acetic acid; TMAO: trimethylamine-N-oxide; CKD: chronic kidney disease; HD: hemodialysis; ↔: no change; ↑: increase; ↓: decrease.
